# Notfälle aus Perspektive der Psychosozialen Akuthilfen – Die Arbeit von Kriseninterventionsteams

**DOI:** 10.1007/s00103-022-03561-8

**Published:** 2022-07-04

**Authors:** Sebastian Hoppe

**Affiliations:** KIT-München, Arbeiter-Samariter-Bund Regionalverband München/Oberbayern e. V., Adi-Maislinger Str. 6–8, 81373 München, Deutschland

**Keywords:** Psychosoziale Notfallversorgung (PSNV), Psychische Erste Hilfe, Krisenintervention im Rettungsdienst, Psychosoziale Unterstützung, Notfallseelsorge, Psychosocial emergency care (PSNV), Psychological first aid, Crisis intervention in rescue services, Psychosocial support, Emergency pastoral care

## Abstract

Teams der Krisenintervention und Notfallseelsorge füllen seit wenigen Jahrzehnten eine Versorgungslücke in der nichtpolizeilichen Gefahrenabwehr. Die Psychosozialen Akuthilfen (PSAH) als Teilbereich der Psychosozialen Notfallversorgung (PSNV) konzentrieren sich auf Angehörige und Hinterbliebene, Vermissende, Augenzeug*innen und Überlebende von belastenden Ereignissen und bieten unmittelbar ereignisbezogene psychosoziale Unterstützung an.

Der Einsatz von Kriseninterventionsteams (KIT) findet inzwischen breite Akzeptanz und Anerkennung: Einsatzkräfte von KIT leisten auf Basis einer fundierten Ausbildung wichtige psychosoziale Unterstützung, die klaren Leitlinien folgt. Qualitätssicherung, gesetzliche Grundlagen und die Frage der Finanzierung von PSAH werden die zentralen Themen des aktuellen Jahrzehnts sein.

Der vorliegende Artikel gibt einen umfassenden Überblick über die Arbeit von KIT und beschreibt die Struktur, Handlungslogik und Ziele der PSAH. Fokus ist dabei die Darstellung der Einsatzabläufe und insbesondere der einzelnen Maßnahmen während der KIT-Betreuungen.

## Abstract (en)

Crisis intervention and emergency counseling teams have been filling a supply gap in non-police emergency response for the past few decades. Psychosocial acute assistance (PSAH) as a subsection of psychosocial emergency care (PSNV) focuses on relatives, people missing someone, and eyewitnesses and survivors of stressful events and offers immediate event-related psychosocial support.

The operations of crisis intervention teams (KIT) are now widely accepted and recognized; KIT emergency services provide important psychosocial support based on profound training following clear guidelines. Quality assurance, legal foundations, and the question of financing PSAH will be the central topics of the present decade.

This article gives a comprehensive overview of the work of KIT and describes the structure, logic of action, and goals of the PSAH. The focus is on the presentation of the operational processes and the individual measures during KIT operations.

## Einleitung und Hintergrund

Als beim Kriseninterventionsteam (KIT) der Alarm eingeht, hat der Rettungsdienst die Reanimation eines etwa 60-jährigen Mannes soeben erfolglos eingestellt. Zuvor war der Familienvater zu Hause zusammengebrochen. Die Alarmierung des KIT erfolgt zur Betreuung von Ehefrau und Tochter des Verstorbenen. Nach einer Übergabe durch Rettungsdienst und Polizei stoßen die beiden KIT-Einsatzkräfte zu den Angehörigen, die in der Küche sitzen. Sie erklären, warum gleich noch ein*e Leichenschauer*in und die Kriminalpolizei kommen werden und was dann weiter passiert. Auch durch das Vermitteln im Kontakt mit der Kriminalpolizei können sie die aufgewühlte Situation beruhigen. Gemeinsam wird besprochen, wie die Mutter dem in Kürze heimkehrenden Sohn die Nachricht überbringen kann. Begleitet durch das KIT nehmen die Angehörigen noch an Ort und Stelle Abschied vom Verstorbenen. Als später mehr Ruhe einkehrt, verabschiedet sich das KIT.

Rettungsdienstliche Versorgung und Gefahrenabwehr beschränken sich nicht (mehr) auf den notfallmedizinischen Bereich. Zunehmend gewinnt auch die psychosoziale Seite von Notfällen an Bedeutung in der Versorgungsstruktur und es wird mittlerweile anerkannt, dass ein Notfall nicht nur erkrankte oder verletzte Personen betrifft. Auch deren unmittelbares Umfeld – ganz gleich, ob Angehörige oder Augenzeug*innen sowie Unfallbeteiligte – muss bedacht und ggf. mitversorgt werden. Die psychosoziale Komponente wird nun systematisch berücksichtigt und insbesondere durch Teams der Krisenintervention und Notfallseelsorge als Bestandteil der Gefahrenabwehr abgedeckt.

Noch vor rund 30 Jahren konnte es passieren, dass Kräfte des Rettungsdienstes etwa nach einer frustranen Reanimation mit Hinterbliebenen im Wohnzimmer standen und nicht wussten, wie sie sich aus dieser unangenehmen Situation lösen konnten. Nicht selten wurde dann verstohlen der Funkmeldeempfänger aus- und wieder eingeschaltet. Bei damaligen Modellen ertönte beim Einschalten der Alarmton, sodass ein weiterer Einsatz vorgetäuscht wurde und man sich zügig verabschieden konnte.

Diese nachvollziehbare Unsicherheit muss sich heute nicht wiederholen: Inzwischen gibt es neben Schulungen des rettungsdienstlichen Personals in psychosozialer Betreuung auch Strukturen speziell zur Betreuung unverletzter Betroffener, die die Einsatzkräfte vor Ort im Bedarfsfall anfordern können.

In den 1990er-Jahren formierten sich zunächst vergleichsweise unstrukturiert und auf Initiative Einzelner erste Kriseninterventions- und Notfallseelsorgeteams. Diese wurden insbesondere durch den vom Bundesamt für Bevölkerungsschutz und Katastrophenhilfe (BBK) moderierten Konsensus-Prozess (2007–2010) standardisiert und qualitativ weiterentwickelt [1]. Neben der kontinuierlichen Einsatzerfahrung im alltagsnahen Bereich führten auch einzelne Großschadenslagen zur strukturellen und qualitativen Weiterentwicklung des Fachbereichs^1^. Das aktuelle Jahrzehnt wird wegweisend sein für die gesetzliche Normierung und damit auch die Finanzierung dieser sogenannten Psychosozialen Akuthilfen (PSAH). Derzeit handelt es sich bei PSAH (noch) um freiwillige Leistungen der Städte und Kommunen. Doch in einigen Bundesländern entstehen derzeit erste gesetzliche Grundlagen [2] und es ist davon auszugehen, dass sich dieser Prozess bundesweit fortsetzen wird. Struktur, Ziele und Arbeitsweise von KIT sollen im folgenden Beitrag dargestellt werden.

## Struktur der Psychosozialen Notfallversorgung

Psychosoziale Unterstützung infolge belastender Ereignisse wird als Psychosoziale Notfallversorgung (PSNV) bezeichnet, die sich in je einen Teilbereich für Einsatzkräfte (PSNV-E) und einen für Betroffene (PSNV-B) unterteilt. PSNV‑E umfasst die Unterstützung von Einsatzkräften (u. a. des Rettungsdienstes, der Feuerwehr und Polizei) im Kontext belastender Einsatzsituationen. Dem Präventionsansatz folgend soll bereits eine angemessene Ausbildung auf belastende Einsätze vorbereiten und die Resilienz stärken (primäre Prävention). Kommt es zu belastenden Einsatzerfahrungen, sollen anlassbezogen weitere Angebote insbesondere im Peer-System längerfristige Folgen, wie z. B. Traumafolgestörungen, verhindern bzw. reduzieren (sekundäre Prävention). Entwickelt sich dennoch eine schwerwiegende psychosoziale Problemlage, wie etwa eine Traumatisierung, ist professionelle Unterstützung indiziert (tertiäre Prävention; [1]).

Die PSNV‑B umfasst die Gesamtstruktur der psychosozialen Versorgung Betroffener. Dazu zählen die sogenannte Psychische Erste Hilfe durch Einsatzkräfte, die PSAH durch Teams der Krisenintervention und Notfallseelsorge sowie längerfristige Unterstützungsangebote. Für Letztere genügt oftmals schon das soziale Umfeld, nötigenfalls können auch niedrigschwellige Beratungsangebote oder in Einzelfällen ärztliche oder psychotherapeutische Hilfen in Anspruch genommen werden [1]. Die Teams der Krisenintervention und Notfallseelsorge werden bereits in der peritraumatischen Phase aktiv, also nur Minuten oder wenige Stunden nach Eintritt bzw. Kenntnisnahme belastender Ereignisse wie unerwarteter Todesfälle oder schwerer Unfälle und sind damit Teil der Gefahrenabwehr. Die Zielgruppe umfasst praktisch alle psychisch Betroffenen eines schwerwiegenden Ereignisses, also Angehörige, Hinterbliebene, Vermissende, Augenzeug*innen und/oder Überlebende [1] – jedoch keine Einsatzkräfte (dies ist Aufgabe der oben skizzierten PSNV-E). Der übergeordnete Begriff PSNV wird oftmals als Synonym für den Teilbereich der PSAH verwendet, was leicht zu inhaltlichen Unschärfen führt und das Verständnis für die strukturell komplexe PSNV zusätzlich erschwert. Dass etwa eine Psychotherapie, die infolge eines belastenden Ereignisses aufgenommen wird, auch (noch) zur PSNV gehört, ist nicht immer intuitiv und wohl vielfach selbst den beteiligten Psychotherapeut*innen nicht bewusst [1].

Wie bereits angedeutet wurde, fehlen bislang in fast allen Bundesländern gesetzliche Grundlagen für die PSAH. Die S2k-Leitlinien „Diagnostik und Behandlung von akuten Folgen psychischer Traumatisierung“ [3] bilden eine wichtige Orientierung zur Arbeit auch in der PSAH. Darüber hinaus gelten die Ergebnisse des genannten Konsensus-Prozesses des BBK [1] als zentral für den Bereich, wenngleich sie lediglich empfehlenden Charakter haben. In vielen Einrichtungen sind die Konsensusempfehlungen bis heute nicht vollständig umgesetzt. Insoweit unterscheiden sich Struktur und Qualität einzelner Teams der PSAH im bundesweiten Vergleich zum Teil deutlich^2^. Die nachfolgenden Ausführungen orientieren sich am Münchener Kriseninterventionsteam „KIT-München“ des Arbeiter-Samariter-Bundes (ASB), können aber auf viele andere Kriseninterventionsteams in gleicher oder ähnlicher Form übertragen werden.

## Einsatzdienst

### Dienstbereitschaft

KIT sind integraler Bestandteil des Rettungsdienstes. Die ehrenamtlich tätigen KIT-Einsatzkräfte verfügen über rettungsdienstlich gekennzeichnete Einsatzfahrzeuge. Vereinzelt ist dabei, je nach lokalen behördlichen Vereinbarungen, die indikationsabhängige Inanspruchnahme von Sonder- und Wegerechten möglich. Diensthabende Einsatzkräfte können sich mit ihrem Fahrzeug frei im jeweiligen Rettungsdienstbereich bewegen, müssen aber zu jedem Zeitpunkt sofort einsatzbereit sein. Eine Hilfsfrist, die die maximale Dauer von Alarmierung bis Eintreffen am Einsatzort definiert, existiert bislang nicht.

Die Einsatzzahlen unterscheiden sich je nach Region deutlich und hängen u. a. maßgeblich von der Vernetzung zwischen PSAH und anderen Einsatzdiensten ab: Nur wenn die Einsatzkräfte von Rettungsdienst, Feuerwehr und Polizei den Bedarf an PSAH erkennen und diese über die Leitstelle anfordern, wird das KIT gerufen (eine Alarmierung des KIT durch Privatpersonen ist nicht vorgesehen). Die Spannweite reicht von gelegentlichen Alarmierungen (insbesondere im ländlicheren Raum) über durchschnittlich 2–3 Alarmierungen einzelner Teams pro Tag bis hin zu 6–7 Alarmen innerhalb von 24 h.^3^

### (Kontra‑)Indikationen

Kennzeichnend für das Indikationsspektrum der Krisenintervention ist eine akute psychische oder psychosoziale Betroffenheit durch eine Konfrontation mit dem plötzlichen Tod (oder der realen Möglichkeit seines Eintretens). Liegt das Ereignis länger als etwa 24–48 h zurück, schließt sich das Zeitfenster der Krisenintervention. Sind Betroffene selbst körperlich verletzt, steht die notfallmedizinische Versorgung im Vordergrund und eine Betreuung im Sinne der PSAH muss hintangestellt werden (sie kann z. B. später durch die Klinikseelsorge geleistet werden). Vorbestehende psychiatrische Erkrankungen sowie akute Selbst- und/oder Fremdgefährdung gelten als Kontraindikation für das KIT – seine Einsatzkräfte sind keine Psychotherapeut*innen oder Ärzt*innen. Entsprechende Fälle übersteigen juristisch und fachlich ihre Kompetenz.

**Figure 1:**
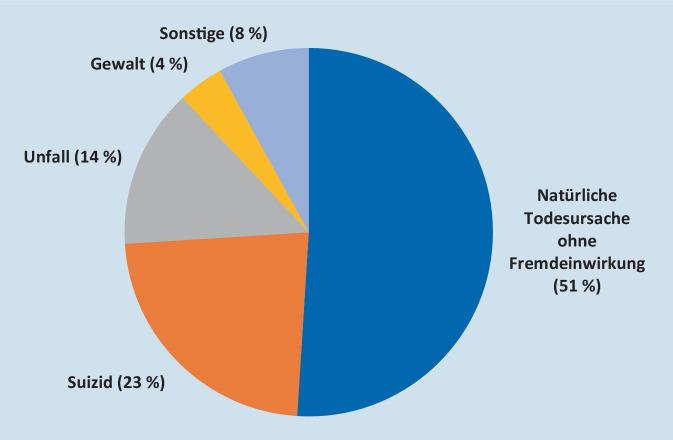

Die Vorstellung, das KIT komme vor allem bei besonders spektakulären Ereignissen, über die in den Medien berichtet wird, bestätigt sich mit Blick auf die Einsatzrealität nicht (Abb. 1). Weitaus häufiger wird Krisenintervention außerhalb der öffentlichen Wahrnehmung tätig, etwa im häuslichen Bereich. Meist liegen natürliche Todesursachen ohne Fremdeinwirkung zugrunde (51 %), oftmals aber auch Suizide (23 %) oder Unfälle (14 %), vereinzelt Gewalttaten (4 %) oder andere Ereignisse (8 %). Immer wieder wird das KIT von der Polizei zum Überbringen einer Todesnachricht hinzugezogen (in etwa 15 % aller Einsätze). Während sich die Polizei nach dem Überbringen der Nachricht und dem Klären organisatorischer Details bald wieder verabschiedet, bringt die KIT-Einsatzkraft Zeit mit – ein Prinzip, das jedem KIT-Einsatz zugrunde liegt und bei der Indikation „Überbringen Todesnachricht“ besonders deutlich wird.

### Zusammenarbeit und Vernetzung

Im Einsatz arbeitet das KIT eng mit Einsatzkräften von Rettungsdienst, Feuerwehr und Polizei zusammen, darüber hinaus je nach Einsatzort auch mit Schulkriseninterventionsteams oder der Klinikseelsorge. Führungskräfte (Leiter*in PSNV sowie ggf. Fachberater*in PSNV) sollen bei komplexeren Schadenslagen die Integration der PSNV in die Einsatzstrukturen sicherstellen. Anlassbezogene Koordinierungsstellen sowie – sofern vorhanden – Landeszentralstellen koordinieren nötigenfalls im Hintergrund [1], auch damit in jedem Setting die angemessenen Versorgungsstrukturen greifen können und nicht übergangen werden.

Zur Überwindung möglicher Sprachbarrieren im Einsatz empfiehlt sich die Vernetzung mit lokalen Dolmetscher*innen-Netzwerken, die im Bedarfsfall unterstützen können. Ebenso selbstverständlich ist die Zusammenarbeit der PSAH mit weiterführenden Hilfsangeboten, etwa Beratungsstellen für Kinder und deren Bezugspersonen oder Geflüchtete aus Kriegsgebieten, – ein Feld, das die PSAH bislang nur unzureichend abdecken kann, das aber aktuell dramatisch an Bedeutung zunimmt.

### Einsatzdokumentation und Psychohygiene

Jeder KIT-Einsatz wird dokumentiert. Seit 2019 steht dazu bundesweit ein standardisiertes Einsatzprotokoll zur Verfügung, das alle Teams der PSAH nutzen können [5].^4^ Diese Dokumentation kann für Einsatzkräfte den logischen Abschluss eines jeden Einsatzes darstellen und bietet die Basis für Auswertungen und Forschung. Weiterhin umfasst das Einsatzprotokoll wichtige Items zu möglichen Belastungen aufseiten der Einsatzkraft und dazu, ob ein Gesprächswunsch mit der Teamleitung besteht. Regelmäßige sowie nötigenfalls auch einsatzbezogene Supervisionsangebote sollen dazu beitragen, dass KIT-Einsatzkräfte ihre Arbeit dauerhaft ausüben können und auch nach für sie selbst belastenden Ereignissen bzw. Einsatzverläufen gesund bleiben.

## Ziele und Maßnahmen der PSAH

**Figure 2:**
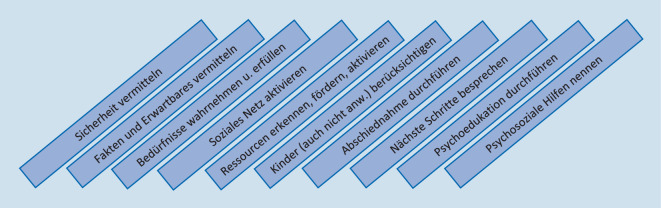

So unterschiedlich das konkrete Ereignis und auch der KIT-Einsatzverlauf sein mögen, im Kern bleibt das Ziel der PSAH immer gleich: Es geht darum, die Handlungsfähigkeit der Betroffenen wiederherzustellen bzw. zu stärken sowie durch die Aktivierung vorhandener persönlicher Ressourcen eine Perspektive für die nächsten Stunden und Tage zu entwickeln. Wie dies konkret erreicht werden kann, orientiert sich maßgeblich am individuellen Bedarf der Betroffenen. Dennoch lässt sich das Vorgehen im Einsatz durch einige wesentliche Maßnahmen zusammenfassen, die in Abb. 2 schematisch dargestellt werden [6]. Grundlage sind die von Hobfoll et al. [7] beschriebenen 5 Prinzipien für Akutinterventionen, die auf der Förderung von Sicherheit, Ruhe, Selbst- und kollektiver Wirksamkeit, Verbundenheit sowie Hoffnung beruhen. Das dargestellte Schema ist dabei lediglich als Orientierungshilfe gedacht und keinesfalls als starrer Ablaufplan zu verstehen. So können einzelne Maßnahmen je nach Situation nicht umsetzbar oder schlichtweg kontraindiziert sein. Die Reihenfolge der Maßnahmen kann variieren.

### Sicherheit vermitteln

Fundament jeder Betreuung ist das Fördern von Sicherheit, so gut dies möglich ist. Erst dann können spätere Maßnahmen ihre Wirkung entfalten, denn eine Betreuung unter (mindestens subjektiv empfundener) Bedrohung ist kaum möglich. So ist ein erster Schritt, Betroffene aus möglichen Gefahrenbereichen herauszuführen und etwa vor Presse oder Schaulustigen zu schützen. Genauso ist es wichtig aufzuzeigen, dass mögliche Gefahren *jetzt* nicht mehr bestehen und z. B. verletzte Personen notfallmedizinisch versorgt werden.

### Fakten und Erwartbares vermitteln

Fast immer ist es für Betroffene von großer Bedeutung, Informationen über das Ereignis oder den aktuellen Gesundheitszustand verletzter/erkrankter Angehöriger zu erhalten. Was ist eigentlich genau passiert? Wie geht es möglichen Verletzten/Erkrankten? In welche Klinik kommt mein Ehemann? Aber auch: Kann ich mein verstorbenes Kind noch einmal sehen? Wo wird es hingebracht? Antworten auf solche und ähnliche Fragen sind in beinahe jeder Betreuung zentral. Aufgabe der PSAH ist es, die notwendigen Informationen im Austausch mit anderen Einsatzkräften und beteiligten Institutionen so gut wie möglich in Erfahrung zu bringen und Betroffenen ehrlich zu kommunizieren. Stehen bestimmte Informationen noch nicht zur Verfügung, sollte den Betroffenen erklärt werden, wo sie diese zu einem späteren Zeitpunkt erfragen können.

### Bedürfnisse wahrnehmen und erfüllen

Die Bedürfnisse von Menschen in bzw. unmittelbar nach belastenden Ereignissen können sehr unterschiedlich sein und sind stark von der jeweiligen Situation abhängig. Neben dem schon angesprochenen Informationsbedürfnis entwickeln Betroffene oftmals ein starkes Redebedürfnis – oder aber im Gegenteil das Bedürfnis nach Ruhe, um sich mit ihren Gedanken und Empfindungen auseinandersetzen zu können. Manchmal ist ein Spaziergang genau das richtige, um die Anspannung und Energie der Belastung körperlich ausagieren zu können. Oftmals sind auch vermeintlich banale Aspekte von Bedeutung: Nicht selten haben Betroffene seit vielen Stunden nichts gegessen oder getrunken. In der kalten Jahreszeit kann schon das beheizte KIT-Einsatzfahrzeug das Grundbedürfnis nach Wärme erfüllen. Kurzum: Einsatzkräfte der Krisenintervention versuchen, die dringlichsten Bedürfnisse zu identifizieren und unterstützen bei deren Erfüllung.

### Soziales Netz aktivieren

Der Grundsatz einer jeden KIT-Betreuung lautet: Wenn sich die Einsatzkraft verabschiedet, soll dafür gesorgt sein, dass Betroffene nicht allein und vielleicht sogar sozial isoliert zurückbleiben. Wo immer möglich, werden Bezugspersonen in Familie, Freundes- oder Bekanntenkreis informiert und zur Unterstützung hinzugezogen. Krisenintervention unterstützt Betroffene dabei, das soziale Netz zu aktivieren. Dabei gelten auch hier die Prinzipien „Hilfe zur Selbsthilfe“ und „Wiederherstellen eigener Handlungsfähigkeit“: Anstatt den Betroffenen notwendige Telefonate abzunehmen, werden diese vielmehr bei der Durchführung unterstützt. Bestehen bei Betroffenen hingegen keinerlei soziale Kontakte (mehr), so stellt dies eine besondere Herausforderung dar, denn soziale Unterstützung gilt als wesentlicher Schutzfaktor im Hinblick auf spätere Belastungen und Traumafolgestörungen [3]. Niedrigschwellige Hilfsangebote sozialer Dienste und Beratungsstellen können hier eine notdürftige, jedoch sehr wichtige mittelfristige Begleitung darstellen.

### Ressourcen erkennen, fördern und aktivieren

In der Betreuung gilt es, mit betroffenen Personen gemeinsam zu identifizieren, was in den nächsten Stunden, Tagen und Wochen hilfreich sein kann. Ideal sind dabei Aktivitäten, die den Betroffenen erfahrungsgemäß guttun: Verabredungen eingehen, Hobbys und Sport ausüben, ein heißes Bad nehmen, laut Musik hören etc. Wie schon bei der vorherigen Maßnahme geht es auch hier in erster Linie darum zu verhindern, dass Betroffene in Passivität oder Isolation abrutschen. Stattdessen sollen sie erkennen, dass das Leben auch künftig lebenswert ist, selbst wenn es sich momentan nicht so anfühlen mag. Der letztgenannte Gedanke ist nicht zu verwechseln mit Floskeln wie: „Die Zeit heilt alle Wunden“ oder „Alles wird gut“. Solche gut gemeinten Sätze können eine große Distanz aufbauen, weil sie u. U. offenbaren, dass das Leid nicht ernstgenommen wird und keine Empathie für die Situation Betroffener besteht. Es gilt, die Dramatik vollständig anzuerkennen – und gleichzeitig behutsam aufzuzeigen, dass es eine Perspektive gibt.

### Kinder (auch nicht anwesend) berücksichtigen

In der Hektik von Notfallereignissen können Kinder – ob gerade anwesend oder nicht – leicht aus dem Fokus geraten und sprichwörtlich „untergehen“. Wichtig ist, ihre Bedürfnisse zu berücksichtigen und mit ihren Bezugspersonen (in der Regel den Eltern) zu besprechen, was im Hinblick auf die Kinder und deren Systeme (Schule, Kindergarten, Freundeskreis) nun wichtig ist. Speziell auf die Unterstützung von Kindern (und deren Bezugspersonen) zugeschnittene Unterstützungsansätze finden sich u. a. im Konzept von Leuchttürmen und Seefahrer*innen von Kern [8] sowie im KASPERLE-Modell nach Karutz [9, 10].

### Abschiednahme durchführen

Fast immer ist es Betroffenen ein tiefes Bedürfnis, verstorbene Angehörige noch einmal zu sehen und sich von ihnen zu verabschieden. Dieses wortwörtliche Begreifen des Todes ist für die Realisierung des Geschehenen und damit auch die Verarbeitung von großer Bedeutung. Die KIT-Einsatzkraft muss die Möglichkeit einer Abschiednahme frühzeitig mit den beteiligten Einsatzkräften abklären. Auch bei Verstorbenen mit schweren äußeren Verletzungen sind Abschiednahmen oftmals möglich und hilfreich. Wichtig ist, Betroffene gut auf die Situation vorzubereiten und die Abschiednahme so würdevoll wie möglich zu gestalten (Material des Rettungsdienstes entfernen, Kerze entzünden, ggf. einzelne Körperteile abdecken etc.).

### Nächste Schritte besprechen

Betroffene sind häufig ratlos, wie es in den nächsten Tagen weitergehen soll. Ergänzend zur oben beschriebenen Ressourcenarbeit ist es wichtig zu besprechen, was in nächster Zeit auch organisatorisch zu erledigen ist (etwa mit Arbeitgeber*in, Schule, Krankenkasse, Vermieter*in, Bestatter*in). Je nach Situation können die einzelnen Schritte schon während der Betreuung näher geplant werden. Manchmal ist dies aber eher zusätzliche Überforderung als entlastende Hilfe, sodass es dann zielführender ist, entsprechende Informationen zu hinterlegen und deutlich zu machen, dass alles Wichtige in den nächsten Tagen in Ruhe und ggf. mit der Unterstützung von Bekannten nachgelesen werden kann. Manche KIT halten dazu eigene Merkblätter vor.

### Psychoedukation durchführen

Infolge eines belastenden Ereignisses nehmen Betroffene oftmals erhebliche Veränderungen oder Reaktionen an sich wahr. Zu den Aufgaben der Krisenintervention gehört es, in einer auch für Laien verständlichen Form aufzuzeigen, dass Veränderungen aus dem Spektrum der akuten Belastungsreaktion normale Reaktionen auf ein nicht normales Ereignis sind und dass in nächster Zeit ggf. mit weiteren Folgen zu rechnen ist. Allerdings kann eine schlecht durchgeführte Psychoedukation^5^ mehr schaden als nützen [3]: Etwa dann, wenn eine sich selbst erfüllende Prophezeiung erzeugt wird, die suggeriert, dass bestimmte Reaktionen zwangsläufig auftreten. Stattdessen sollte deutlich gemacht werden, dass die Verarbeitung belastender Ereignisse keine lineare Entwicklung ist, mögliche auftretende Reaktionen aber zumeist über die nächsten Tage und wenige Wochen nachlassen. Auch hier kann eng an die oben beschriebene Ressourcenarbeit angeknüpft werden, um positive Bewältigungsstrategien aufzuzeigen. Für den Fall, dass die erwartete Linderung nicht eintritt, sollten Hilfsangebote genannt werden. Beispielhaft für die Psychoedukation sei auf den entsprechenden Flyer des BBK [11] sowie die auf der Website der Psychotherapeutenkammer Niedersachsen [12] in mehreren Sprachen zur Verfügung gestellten Informationsmaterialien verwiesen. Letztgenannte Materialien entstammen einem vom National Child Traumatic Stress Network (NCTSN) sowie dem National Center for Posttraumatic Stress Disorder (NCPTSD) veröffentlichten Manual zur Psychischen Ersten Hilfe [13]. Erstellt und evaluiert durch ein Expert*innengremium wird es seit mehreren Jahrzehnten weltweit genutzt. Eine deutsche Adaption erfolgte durch Kröger [4].

### Psychosoziale Hilfen nennen

In bestimmten Fällen kann es wichtig und hilfreich sein, professionelle Unterstützung in Anspruch zu nehmen. Dass niemand mit belastenden Ereignissen allein zurechtkommen muss und die Inanspruchnahme von Hilfe kein Zeichen von Schwäche ist, sollte in der Betreuung deutlich werden. Dabei dürfen Betroffene nicht pathologisiert werden, also den Eindruck bekommen, sie seien krank. Sofern nötig, können konkrete Hilfsangebote genannt werden, etwa von Beratungsstellen, Selbsthilfegruppen oder anderen Institutionen. Je weniger sich Betroffene in der Akutsituation merken müssen und je mehr Informationen nachzulesen sind, desto besser. Deshalb sind auch hier Flyer der jeweiligen Institutionen hilfreich.

## Haltung und Selbstverständnis

KIT stellen ein niedrigschwelliges aufsuchendes *Angebot* in der Akutphase dar, das abgelehnt werden darf: PSAH drängen sich nicht auf. Betroffene sind nicht per se „krank“, sondern waren oder sind lediglich einem belastenden Ereignis ausgesetzt. Es gilt, Betroffene in der Betreuung nicht zu bevormunden, sondern sie vorbehaltlos bei dem zu unterstützen, was für sie in dieser Situation wichtig ist. Die KIT-Einsatzkraft bringt Zeit mit, nimmt wahr und hält mit aus. Neben diesem buchstäblichen Dasein geht es außerdem darum, gemeinsam mit der betroffenen Person vorzubereiten, wie es weitergehen kann, wenn sich das KIT wieder verabschiedet hat. Je eher Betroffene wieder selbst handlungsfähig und nicht auf weitere akute Unterstützung angewiesen sind, desto besser. Zudem ist Krisenintervention zeitlich wie inhaltlich klar begrenzt: Die grundsätzlich nur einmalige Betreuung bezieht sich immer auf ein klar eingrenzbares Akutereignis im Kontext des plötzlichen Todes oder seiner realen Möglichkeit. Etwaige tieferliegende Belastungen und Probleme, wie etwa langjährige Familienkonflikte oder vorbestehende psychische Erkrankungen, können in der Krisenintervention selbstverständlich nicht aufgearbeitet werden.

## Ausbildung und Forschung

Einsatzkräfte der PSAH durchlaufen zunächst eine theoretische Ausbildung, die etwa 110 Unterrichtseinheiten umfasst. Hilfsorganisationen und Kirchen haben dazu gemeinsame Mindeststandards definiert [14], die aber wegen der fehlenden Normierung der PSAH eher empfehlenden als verbindlichen Charakter haben. Immerhin – und das ist einer der zentralen Erfolge des eingangs erwähnten Konsensus-Prozesses des BBK: Wer entgegen den geltenden Leitlinien und auch den Mindeststandards der Ausbildung handelt, bringt sich in Erklärungsnot und wird kaum Anerkennung finden.

Der sich anschließende praktische Teil der Ausbildung unterscheidet sich von Team zu Team. Wünschenswert ist, dass die Einsatzkräfte in Ausbildung im Verlauf etwa eines Jahres rund 10–20 Einsätze absolvieren, in denen sie erfahrene Kolleg*innen begleiten, unterschiedliche Meldebilder kennenlernen und zunehmend mehr Verantwortung übernehmen. Mit abgeschlossener Ausbildung wird ein PSNV-Ausweis ausgehändigt, der die Befugnisse regeln und vor selbsternannten Helfer*innen schützen soll.

Das junge Gebiet der PSAH ist bislang kaum wissenschaftlich erforscht, was insbesondere an den großen Herausforderungen bei der Konstruktion entsprechender Forschungsdesigns liegt [3] und sukzessive angegangen werden sollte.

## Fazit und Ausblick

Mit ihrer psychosozialen Unterstützung für Menschen unmittelbar nach außergewöhnlich belastenden Ereignissen füllen KIT als Teil der PSAH und damit der PSNV seit wenigen Jahrzehnten eine eklatante Versorgungslücke in der Gefahrenabwehr. Alarmiert von Einsatzkräften des Rettungsdienstes, der Feuerwehr und der Polizei ist das KIT für Betroffene da, bringt Zeit mit, hält die belastende Situation mit aus und unterstützt bei praktischen Fragen.

Während gesetzliche Grundlagen und damit auch eine Finanzierung sowie verbindlich geltende Richtlinien dringend benötigt und belastbare Ergebnisse der Wirksamkeitsforschung für die nächsten Jahre erwartet werden, steht schon seit Langem fest: Menschen dürfen infolge belastender Ereignisse nicht sich selbst überlassen werden und KIT leisten dabei eine zentrale Aufgabe.

## Footnotes

1 Hoppe S (2016) Der Einfluss des Konsensus-Prozesses auf die Praxis der Psychosozialen Notfallversorgung für Betroffene (PSNV-B). Vergleichende Darstellung anhand der Einsatzbeispiele Eschede 1998 und Bad Aibling 2016. Unveröffentlichte Masterthesis, International Psychoanalytic University Berlin.

2 Hoppe S (2016) Der Einfluss des Konsensus-Prozesses auf die Praxis der Psychosozialen Notfallversorgung für Betroffene (PSNV-B). Vergleichende Darstellung anhand der Einsatzbeispiele Eschede 1998 und Bad Aibling 2016. Unveröffentlichte Masterthesis, International Psychoanalytic University Berlin.

3 Die hier sowie im nachfolgenden Abschnitt genannten Einsatzzahlen beziehen sich auf das KIT-München im Jahr 2021 (unveröffentlichte Jahresstatistik).

4 Von Kröger [4] steht seit 2013 eine in grundlegenden Aspekten ähnliche Einsatzdokumentation zur Verfügung, die sich in zahlreichen Details jedoch wesentlich unterscheidet.

5 Der Begriff Psychoedukation wird im Kontext der PSAH seit vielen Jahren kontrovers diskutiert, da er im engeren Sinne heilkundlichem Handeln vorbehalten ist. Alternativ wird auch von Psychoinformation gesprochen. Der Autor folgt den S2k-Leitlinien, in denen der Begriff der Psychoedukation verwendet wird [3].
